# Using a generalized additive model with autoregressive terms to study the effects of daily temperature on mortality

**DOI:** 10.1186/1471-2288-12-165

**Published:** 2012-10-30

**Authors:** Lei Yang, Guoyou Qin, Naiqing Zhao, Chunfang Wang, Guixiang Song

**Affiliations:** 1 Department of Biostatistics, School of Public Health, Fudan University, Shanghai, China; 2 Vital Statistics Division, Shanghai Municipal Center for Disease Control & Prevention, Shanghai, China

## Abstract

**Background:**

Generalized Additive Model (GAM) provides a flexible and effective technique for modelling nonlinear time-series in studies of the health effects of environmental factors. However, GAM assumes that errors are mutually independent, while time series can be correlated in adjacent time points. Here, a GAM with Autoregressive terms (GAMAR) is introduced to fill this gap.

**Methods:**

Parameters in GAMAR are estimated by maximum partial likelihood using modified Newton’s method, and the difference between GAM and GAMAR is demonstrated using two simulation studies and a real data example. GAMM is also compared to GAMAR in simulation study 1.

**Results:**

In the simulation studies, the bias of the mean estimates from GAM and GAMAR are similar but GAMAR has better coverage and smaller relative error. While the results from GAMM are similar to GAMAR, the estimation procedure of GAMM is much slower than GAMAR. In the case study, the Pearson residuals from the GAM are correlated, while those from GAMAR are quite close to white noise. In addition, the estimates of the temperature effects are different between GAM and GAMAR.

**Conclusions:**

GAMAR incorporates both explanatory variables and AR terms so it can quantify the nonlinear impact of environmental factors on health outcome as well as the serial correlation between the observations. It can be a useful tool in environmental epidemiological studies.

## Background

People have long been interested in potential impacts of temperature on human health [1-3]. Recently, many studies have been done to analyse the way and the extent temperature influences health outcomes [4-6]. Such studies have practical value for the following reasons: knowing the association between environmental factors and health outcomes will help to identify at-risk populations, benefit health department in resource allocation, and provide support for stake holders in prevention [7].

Environmental epidemiology, the investigation of the health risks related to environmental exposures, becomes the main research approach [8]. There are various study designs in environmental epidemiology for estimating health effects of temperature. One of them is model evaluation of time series data to quantify the association between temperature (or other weather/environmental factors etc.) and daily mortality (or hospitalization etc.). These are a type of ecologic study because they analyse daily population-averaged health outcomes and exposure levels [9]. This approach is suitable to study transient acute effects which are due to time-varying exposures [10-14], and the choice of model can have a large influence on the interpretation of environmental effects.

Generalized Linear Model (GLM) [15] and Generalized Additive Model (GAM) [16] are the main models used in environmental epidemiology. When the response variable represents counts (e.g. number of deaths), the model often takes the form of a Poisson regression/additive model with a log link function. The outcome *Y*_*t*_ is assumed to follow a Poisson distribution with mean *μ*_*t*_ which is linked either to a linear combination (in this case, the model is a GLM) or smoothed functions (in this case, the model is a GAM) of environmental explanatory variables via a log function.

Many studies have identified the nonlinear relations (U, J, or V shaped) of daily mortality on temperature [17-24], which are mainly represented by piecewise linear terms [18-20] or natural cubic splines [21-24]. An assumption is often made that temperature only affects the mortality of the same day or on a single day of lag l [18,20-22], and denoted by the term *temp*_*t-l*_ in the model. When l=0, it represents the effect on the same day [18,20-23], when l>0, it represents a lagged effect [18,20,21,23]. An alternative to single lag models is a distributed lag model [25]. It includes multiple lags of temperature over a period of time based on the assumption that the effect of time is distributed over several days into the future. In all the above models, a spline function of time is often used to explain the long time trend, and confounding effects like humidity and air pollution are also controlled by splines.

However, a thorough time series analysis should consider the order of data points and correlation of adjacent points in time [26]. In environmental epidemiological studies, the response variable may also be correlated and it is necessary to embody autocorrelation of the response variable when modelling. But all the above models are standard GLM/GAM that describes how the response variable is stochastically related to explanatory variables without considering how the response can be dependent also on its past values.

In addition, autocorrelation causes trouble in estimation of GLM/GAM, since GLM/GAM essentially requires each observation to be independently distributed. Violation of this assumption can lead to problematic estimates even in very simple cases. For example, if the error terms in a linear regression model are in reality positively autocorrelated, failure to account for this may underestimate the standard errors of the estimated regression coefficients [27].

Another statistical issue often encountered is overdispersion, and many sources of autocorrelation are related to sources of overdispersion [28]. One usual approach to adjust for overdispersion is to specify a dispersion parameter *ϕ* for estimation [15]. However, if autocorrelation exists, this approach only inflates the variance of the estimate by *ϕ* but leaves the estimate unchanged, which is an inadequate solution to our issue.

Generalized Estimating Equations (GEE) [29,30] and Generalized Linear Mixed Model (GLMM)/Generalized Additive Mixed Model (GAMM) [31,32], are two extensions from GLM/GAM for grouped or clustered data. GEE and GLMM/GAMM can be implemented in popular software like SAS and R [31-34]. For all these models, a within-group variance-covariance structure can be used to account for the corresponding within-group autocorrelation [31,34]. Time series data, treated as single cluster data, can also be modelled by GAMM. Performance of this degenerative GAMM will be studied in simulation.

In this article, we introduced GAM with Autoregressive terms (GAMAR), which is derived from Generalized Autoregressive Moving Average (GARMA) models [35]. ARMA is a family of models for analysing time series. The notation ARMA(p,q) refers to a model with p autoregressive terms and q moving-average terms [26]. GARMA belongs to the class observation driven models [36] for extending Gaussian ARMA to non-Gaussian settings. GAMAR differs from GARMA in that the linear components in GARMA are generalized to natural splines and MA terms are omitted. The generalization is motivated by modelling nonlinear relationships, while the simplification is justified because AR can be used to approximate MA or ARMA. Additionally, larger AR order in GAMAR will not compromise the estimation of the effects of explanatory variables.

In all, GAMAR has two advantages over GAM: 1) it is a model for generalized time series analysis rather than a probabilistic model like GAM; 2) the AR part of GAMAR can explain the autocorrelation structure of observations. So the Pearson residuals of GAMAR can be closer to white noise than those from GAM, yielding more reliable estimation.

## Methods

### GARMA

In GARMA, the conditional distribution of each observation *y*_*t*_, for *t=1,…,n*, given the previous information set *H*_*t*_*={X*_*1*_*,…,X*_*t*_*,y*_*1*_*,…,y*_*t-1*_} containing past observations *y*_*t*_ and covariate vectors *X*_*t*_ = (*X*_*t1*_*,…,X*_*tm*_), is assumed to follow the same exponential family distribution [35]. As with the standard GLM, the conditional mean *μ*_*t*_ is related to the variables by a twice-differentiable one-to-one monotonic function g, which is called the link function. However, unlike the standard GLM, the formula here allows autoregressive moving average terms to be included additively [35]:

$$\begin{array}{l} g\left(\mu_{t}\right)=\sum_{i=1}^{m}X_{\mathrm{ti}}\beta_{i}+\sum_{j=1}^{p}c_{j}\left(g\left(y_{t-j}\right)-\sum_{i=1}^{m}X_{t-j,i}\beta_{i}\right)\\ \;+\sum_{j=1}^{q}d_{j}\left(g\left(y_{t-j}\right)-g\left(\mu_{t-j}\right)\right)\text{,} \end{array}$$

where $\sum_{j=1}^{p}c_{j}\left(g\left(y_{t-j}\right)-\sum_{i=1}^{m}X_{t-j,i}\beta_{i}\right)$ are autoregressive terms and $\sum_{j=1}^{q}d_{j}\left(g\left(y_{t-j}\right)-g\left(\mu_{t-j}\right)\right)$ are moving average terms.

Count data are often assumed to follow Poisson distribution. And the Poisson GARMA submodel is:

$$\begin{array}{l} \ln\left(E\left(y_{t}\right)\right)=\ln\left(\mu_{t}\right)=\sum_{i=1}^{m}X_{\mathrm{ti}}\beta_{i}\\ \;+\sum_{j=1}^{p}c_{j}\left(\ln\left(y_{t-j}^{*}\right)-\sum_{i=1}^{m}X_{t-j,i}\beta_{i}\right)\\ \;+\sum_{j=1}^{q}d_{j}\left(\ln\left(y_{t-j}^{*}/\mu_{t-j}\right)\right)\text{,} \end{array}$$

where *y*_t_^*^ = max(*y*_*t*_*,τ*), *τ* is a positive threshold parameter. Any 0 or negative values of *y* are replaced by *τ*, because ln() is not defined for 0 or negative values.

### GAMAR

GAMAR is derived from GARMA with linear terms replaced by smoothers and MA terms omitted:

(1) $$\begin{array}{l} g\left(E\left(y_{t}\right)\right)=g\left(\mu_{t}\right)=\sum_{i=1}^{m}s_{i}\left(X_{\mathrm{ti}}\right)\\ \;+\sum_{j=1}^{p}c_{j}\left(g\left(y_{t-j}\right)-\sum_{i=1}^{m}s_{i}\left(X_{t-j,i}\right)\right)\text{,} \end{array}$$

where $\sum_{i=1}^{m}s_{i}\left(X_{\mathrm{ti}}\right)$ are smoothers of covariates, $\sum_{j=1}^{p}c_{j}\left(g\left(y_{t-j}\right)-\sum_{i=1}^{m}s_{i}\left(X_{t-j,i}\right)\right)$ are autoregressive terms.

Compared to GAM, (1) allows autoregressive terms to be included additively in the link predictor.

For count data y, we use the Poisson submodel:

(2) $$\begin{array}{l} \ln\left(E\left(y_{t}\right)\right)=\ln\left(\mu_{t}\right)=\sum_{i=1}^{m}s_{i}\left(X_{\mathrm{ti}}\right)\\ \;+\sum_{j=1}^{p}c_{j}\left(\ln\left(y_{t-j}^{*}\right)-\sum_{i=1}^{m}s_{i}\left(X_{t-j,i}\right)\right)\text{,} \end{array}$$

here *y*_t_^*^ = max(*y*_*t*_*,τ*), *τ* is a positive threshold parameter.

In our study, we use natural cubic spline (*ns*) as smoother. *ns* is a piecewise-cubic real function on an interval *a,b*, where *a,b* is separated by a sequence of k ordered knots: *α = ξ*_*0*_*< ξ*_*1*_*< ⋯ < ξ*_*k-1*_*< ξ*_*k*_*= b*. *ns* is continuous at interior knots and linear beyond the boundary [16]. Degrees of freedom (*df*) for *ns* equals to the number of subintervals separated by these knots, thus *df* satisfies: *df = k*. *ns* is often represented by linear combination of its B-spline basis [16]. When *df* is specified, the standard practice is to place *df-1* interior knots at evenly spaced intervals in the data to generate the B-spline basis. By using *ns* as smoother in (2), the Poisson GAMAR becomes:

$$\begin{array}{l} \ln\left(E\left(y_{t}\right)\right)=\ln\left(\mu_{t}\right)=\sum_{i=1}^{m}ns\left(X_{\mathrm{it}},df_{i}\right)\\ \;+\sum_{j=1}^{p}c_{j}\left(\ln\left(y_{t-j}^{*}\right)-\sum_{i=1}^{m}ns\left(X_{i-j,t},df_{i}\right)\right)\text{.} \end{array}$$

### Algorithm

#### Maximum partial likelihood estimator (MPLE)

For the jointly distributed time series {*X*_*t*_*,y*_*t*_}, *t = 1,…n*, the parameters of GAMAR can be estimated by maximum partial likelihood. The partial likelihood based on *y*_*t*_ for {*X*_*t*_*,y*_*t*_}, *t = 1,…n* can be expressed as the product of a sequence of conditional likelihoods *f*(*y*_*t*_*| X*^*(t)*^*,y*^*(t-1)*^*;θ*), *t = 1,…n*, where *X*^*(t)*^*= X*_*1*_*,…X*_*t*_, *y*^(*t-1*)^ = *y*_1_,…*y*_t-1_, that is:

$$PL=\prod_{t=1}^{n}f\left(y_{t}|X^{\left(t\right)},y^{\left(t-1\right)};\theta \right)\text{.}$$

This function is referred to as a partial likelihood instead of likelihood because *X*_*t*_ are stochastic [35]. For a Poisson GAMAR, the partial likelihood is:

$$PL=\prod_{t=1}^{n}\frac{\mu_{t}^{y_{t}}}{y_{t}!}e^{-\mu_{t}}\text{,}$$

and *μ*_*t*_ can be expanded as $\ln\left(\mu_{t}\right)=\sum_{i=1}^{m}ns\left(X_{\mathrm{ti}},df_{i}\right)+\sum_{j=1}^{p}c_{j}\left(\ln\left(y_{t-j}^{*}\right)-\sum_{i=1}^{m}ns\left(X_{t-j,i},df_{i}\right)\right)$. Since a natural cubic spline is a linear combination of its B-spline basis, it is linear with respect to its parameters, so natural cubic spline functions are like linear terms from the perspective of computation. Therefore,

(3) $$\begin{array}{l} \ln\left(\mu_{t}\right)=\eta_{t}=\sum_{i=1}^{m}\beta_{i}X_{\mathrm{ti}}\\ \;+\sum_{j=1}^{p}c_{j}\left(\ln\left(y_{t-j}^{*}\right)-\sum_{i=1}^{m}\beta_{i}X_{t-j,i}\right) \end{array}$$

where *θ* = (*β*_*1*_⋯*,β*_*m*_*,c*_*1*_,⋯,*c*_*p*_)^*T*^ is the model parameter vector.

### Modified Newton’s method

Maximum partial likelihood estimators are solved by modified Newton’s method. This procedure is described in detail in Appendix.

### Evaluation of GAMAR by simulation

We conducted two simulation studies, each with 1000 samples, to compare the performance of GAM and GAMAR for estimation. In simulation study 1, the performance of GAMM is also studied. The R code for simulations and simulation output is included in Additional file 1.

### Simulation study 1

The first model:

(4) $$\begin{array}{l} Y_{t}\sim Poisson(\mu_{t})\\ \ln(\mu_{t})=ns(x_{t},5)+a_{t}\\ ns(x_{t},5)=\sum_{i=1}^{5}\beta_{i}s_{i5}\left(x_{t}\right)\\ a_{t}=\sum_{i=1}^{3}c_{i}\left(\ln\left({y^{*}}_{t-i}\right)-ns\left(x_{t-i},5\right)\right)\\ y_{t}^{*}=\max(y,\tau ),\tau =\text{0.5.} \end{array}$$

Here *x*_*t*_ represents a daily averaged temperature series from year 2000–2008 obtained from Shanghai's Meteorological Bureau, and the terms *s*_*i5*_(*x*_*t*_), *i* = 1, 2, 3, 4, 5 form the B-spline basis for the natural cubic spline. The parameters are:

$$\begin{array}{l} \beta_{0}=5.02,\beta_{1}=-0.35,\beta_{2}=-0.36,\beta_{3}=-0.38,\beta_{4}\\ \;=-0.33,\beta_{5}=-0.15\;\text{and}\;c_{1}=0.5,c_{2}=0.25,c_{3}\\ \;=0.12\text{.} \end{array}$$

We used data ranging over time points 1828–3288 (year 2005–2008) to eliminate the impact of the starting value, where the time points stand for the days since Jan 1st 2000.

For two models on Sample 1, the Autocorrelation Function (ACF) and Partial Autocorrelation Function (PACF) [26] of the Pearson residuals [15] are plotted against different lag periods to examine the presence of residual autocorrelation. Estimates of temperature effects from the two models and the true effect were plotted against temperature to show which model provided a better fit.

Besides showing the analysis for a single sample, the averaged results from GAM, GAMM and GAMAR were given, and the statistics calculated were mean estimates, bias, relative error and coverage, short for “coverage rate of confidence interval (CI) on true value”. The confidence level was chosen to be 95%, so the coverage of a correct model should be around 95%.

In addition, GAMAR with various AR orders were studied to explore the effects of AR order on estimation. Finally, to verify the consistency of partial maximum likelihood estimation, 1000 samples in the time range of 2558–3288 (2 years), 1828–3288 (4 years), 1097–3288 (6 years) were used for calculation.

### Simulation study 2

A second simulation study was performed to study whether the proposed method could approximate a nonlinear curve. We simulated 1000 samples from a model where the covariate is a cosine function of temperature and nonlinear with respect to the parameters.

(5) $$\begin{array}{l} Y_{t}\sim \text{Poisson}(\mu_{t})\\ \ln(\mu_{t})=4.8+0.2\cos(\pi (x_{t}+3)/28)+a_{t}\\ a_{t}=\sum_{i=1}^{3}c_{i}\left(\ln\left(y_{t-i}^{*}\right)-\left(4.8+0.2\cos\left(\pi \left(x_{t}+3\right)/28\right)\right)\right)\\ y_{t}^{*}=\max(y,\tau ),\tau =0.5 \end{array}$$

Here, the AR terms parameters are identical to (4): *c*_1_ = 0.5, *c*_2_ = 0.25, *c*_3_ = 0.12.

As in simulation study 1, sample statistics were calculated over the time points 1828–3288 (year 2005–2008) for each of the 1000 realizations by GAM and GAMAR. The first sample was analysed just the same way as in simulation study 1, but here the mean and standard deviation of the parameter estimates from the two models for the 1000 samples are presented. To compensate for the absence of true parameters, we compared the two models visually, plotting the mean predicted values from the two models and the true effect. Moreover, the Pearson correlation coefficients of the fitted linear predictors from the two models and true effect were also calculated.

### Application to temperature-mortality research

The real example came from three sources: daily mortality of Shanghai in 2001–2004 from Shanghai Municipal Center for Disease Control & Prevention; daily average temperature and humidity in 2001–2004 from Shanghai's Meteorological Bureau; and air pollution data (NO_2_, SO_2_, PM10) in 2001–2004 from (http://www.envir.gov.cn/airnews/), which was announced by the Shanghai Environmental Monitoring Centre.

Autocorrelation of the Pearson residuals from the two models are compared via figure. We also compared their parameter estimates, and their estimated temperature effects via figures. The R code for real example modelling and its outputs is included in Additional file 1.

## Results

### Simulation results

#### Simulation study 1

**Figure 1:**
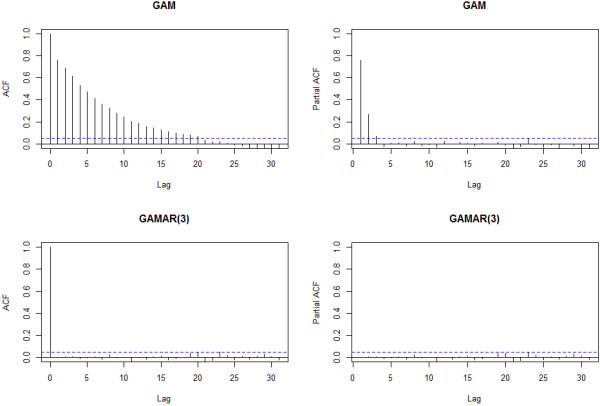
**ACF and PACF of GAM and GAMAR(3) for Sample 1 from simulation study 1.** These statistics are calculated on Pearson residuals $z_{t}=\frac{{\hat{y}}_{t}-y_{t}}{\sqrt{{\hat{y}}_{t}}}$ from both models.

The ACF and PACF plot of the GAM Pearson residuals from the first sample indicate obvious autocorrelation (Figure 1). The Pearson estimate of the dispersion parameter [37] is $\hat{\phi }=2.675>1$, indicating data overdispersion. Since the ACF tails off and PACF cuts off after lag 3, it is natural to use GAMAR(3) instead. The model is given below:

(6) $$\begin{array}{l} \ln(E(y_{t}))=ns(x_{t},5)+\sum_{i=1}^{3}c_{i}\left(\ln\left({y^{*}}_{t-i}\right)-ns\left(x_{t-i},5\right)\right)\\ y_{t}^{*}=\max(y,\tau ),\tau =0.5 \end{array}$$

**Figure 2:**
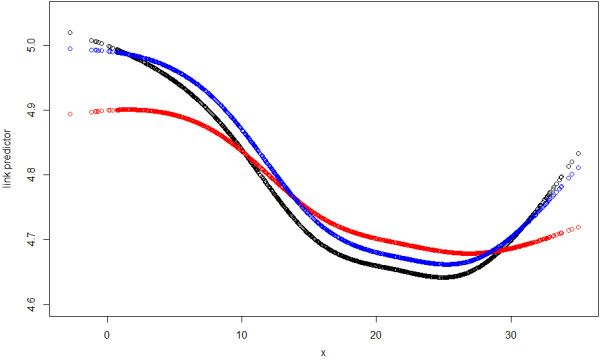
**The temperature effects in link scale for Sample 1 from simulation study 1.** Black: the true effect, Red: $\hat{ns\left(x,5\right)}$ from GAM, Blue: $\hat{ns\left(x,5\right)}$ from GAMAR(3).

The ACF and PACF plot of the GAMAR(3) Pearson residuals suggest no autocorrelation, and the dispersion parameter estimate is now $\hat{\phi }=1.0219\approx 1$, indicating no overdispersion. Apparently, GAMAR(3) controls autocorrelation and overdispersion simultaneously, and Figure 2 indicates that the predicted spline function nsx,5^ from GAMAR(3) is much closer to the real model than that from GAM.

Concerning the general performance of GAM and GAMAR, Table 1 shows that the biases of the mean parameter estimates from GAM are almost the same as GAMAR. However, the coverages of the 95% confidence intervals from GAM are far less than 95%, while those from GAMAR are very close to 95%. Also, relative errors of the parameter estimates from GAM are larger than that of GAMAR.

**Table 1:** Results from GAM, GAMM, GAMAR(3) in simulation study 1

		**GAM**					**GAMM (AR(3) correlation structure)**				**GAMAR(3)**			
	**TruPar**	**MeaEst**	**Bias**	**RelErr**	**Coverage**	**Coverage2**	**MeaEst**	**Bias**	**RelErr**	**Coverage**	**MeaEst**	**Bias**	**RelErr**	**Coverage**
*β*_0_	5.02	4.9991	−0.0209	0.0127	38	58.8	5.0065	−0.0135	0.0049	92	5.0182	−0.0018	0.0055	94.8
*β*_1_	−0.35	−0.3574	−0.0074	0.1922	33.5	52.4	−0.3576	−0.0076	0.0757	88	−0.3561	−0.0061	0.0686	93.9
*β*_2_	−0.36	−0.3672	−0.0072	0.229	35.3	56.4	−0.3662	−0.0062	0.0843	96	−0.362	−0.002	0.0885	94.6
*β*_3_	−0.38	−0.382	−0.002	0.1531	34.2	55.3	−0.3787	0.0013	0.0608	96	−0.3757	0.0043	0.0733	93.8
*β*_4_	−0.33	−0.3281	0.0019	0.428	38.6	62.6	−0.3418	−0.0118	0.1329	98	−0.3262	0.0038	0.1604	95.5
*β*_5_	−0.15	−0.1495	0.0005	0.4924	35.4	55.0	−0.1581	−0.0081	0.202	92	−0.1472	0.0028	0.2201	93.8
	Mea_co		0.0067	0.2512	35.8	56.8		–0.0077	0.0934	93.7		0.0035	0.1027	94.4
*c*_1_	0.5										0.4982	−0.0018	0.0422	95.3
*c*_2_	0.25										0.2477	−0.0023	0.0947	93.4
*c*_1_	0.12										0.1197	−0.0003	0.1737	94.7
	Mea_ar											0.0015	0.1035	94.5

TruPar=True Parameters= *β*_*i*_ or *c*_*i*_.

MeaEst=Mean estimates= ${\overset{―}{\hat{\beta }}}_{i}$ or ${\overset{―}{\hat{c}}}_{i}$.

Bias= ${\overset{―}{\hat{\beta }}}_{i}-\beta_{i}$ or ${\overset{―}{\hat{c}}}_{i}-c_{i}$.

RelErr=Relative Error= $\overset{―}{\left|\left({\hat{\beta }}_{i}-\beta_{i}\right)/\beta_{i}\right|}$ or $\overset{―}{\left|\left({\hat{c}}_{i}-c_{i}\right)/c_{i}\right|}$.

Coverage: the percentage ofestimated 95%CI whichcovers true coefficient in all estimated 95%CI.

Coverage2: coverage from GAM which accounts for overdispersion.

Mea_co: Mean absolute Bias, RelErr, Coverage for parameters of covariates.

Mea_ar: Mean absolute Bias, RelErr, Coverage for parameters of AR terms.

Coverages for GAM corrected for overdispersion [15] are also shown in Table 1 as coverage2. This approach is the same as a standard GAM except that its standard error has been expanded by the dispersion parameter *ϕ*. As a result, the bias and relative errors remain unchanged, while the confidence interval is broadened, coverage improved. However, the coverage is still far less than 95%.

The results of GAMM with AR(3) correlation structure and GAMAR(3) are very similar, but the former model is much more time consuming. This disparity will be described in discussion. Hence results from only 50 samples fitted by GAMM are summarized in Table 1.

To examine how the AR order in GAMAR influences the results, we also fitted the GAMAR(1), GAMAR(2), GAMAR(4), and GAMAR(5). As Table 1 in Additional file 2 shows, the coverage grows and relative error drops as the AR order increases from 1 to 3. For models with AR order larger than 3, there is little difference in coverage, relative error and bias among them.

The results for different time ranges are listed in Table 2 in Additional file 2, from which we can see that the larger the number of time-points, the lower the bias and relative errors, and the higher the coverage. This illustrates the asymptotic unbiased (consistency) property of MPLE.

**Table 2:** Results from GAM and GAMAR(3) in simulation study 2

		**GAM**		**GAMAR(3)**			
	**TruPar**	**MeaEst**	**Sd**	**MeaEst**	**Sd2**	**DifEst**	**DifSd**
		5.006	0.0842	5.0246	0.0371	−0.0186	0.0471
		−0.2812	0.0864	−0.2806	0.0307	−0.0006	0.0557
		−0.3894	0.1074	−0.3859	0.0412	−0.0035	0.0662
		−0.4379	0.1011	−0.4321	0.0399	−0.0058	0.0612
		−0.3975	0.0825	−0.3888	0.0407	−0.0087	0.0418
		−0.4536	0.1886	−0.4442	0.0754	−0.0094	0.1132
		−0.2757	0.1001	−0.2646	0.0448	−0.0111	0.0553
*c*_1_	0.5			0.4978	0.0260		
*c*_2_	0.25			0.2482	0.0287		
*c*_3_	0.12			0.1196	0.0261		

TruPar=True Parameters= *c*_*i*_.

MeaEst=Mean estimates= ${\overset{―}{\hat{\beta }}}_{i}$ or ${\overset{―}{\hat{c}}}_{i}$.

Sd= Standard deviation of 1000 $\hat{\beta }$ and ${\hat{c}}_{i}$.

DifEst=MeaEst of GAM- MeaEst of GAMAR(3).

DifSe=Sd of GAM-Sd of GAMAR(3).

#### Simulation study 2

**Figure 3:**
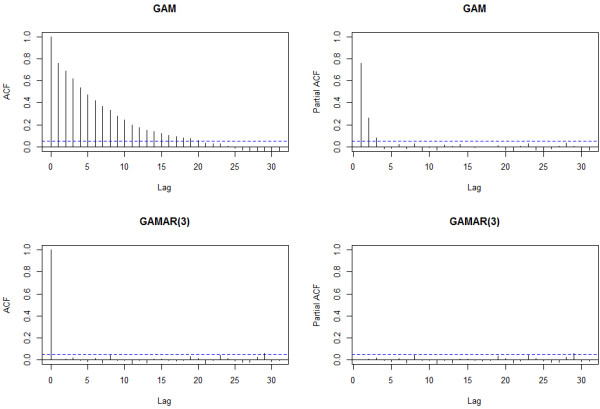
**ACF and PACF of GAM and GAMAR(3) for Sample 1 from simulation study 2.** These statistics are calculated on Pearson residuals $z_{t}=\frac{{\hat{y}}_{t}-y_{t}}{\sqrt{{\hat{y}}_{t}}}$ from both models.

**Figure 4:**
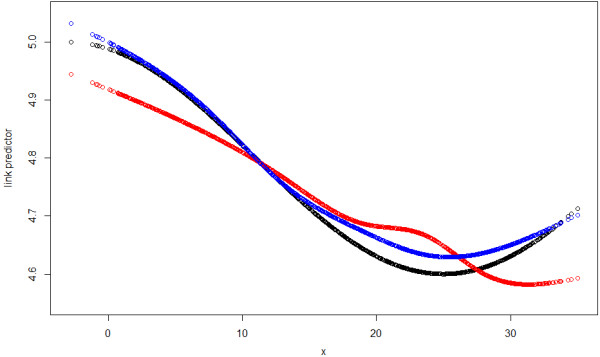
**The temperature effects in link scale for Sample 1 from simulation study 2.** Black: the true effect, Red: $\hat{ns\left(x,6\right)}$ from GAM, Blue: $\hat{ns\left(x,6\right)}$ from GAMAR(3).

In this simulation study, The ACF and PACF plot of the GAM Pearson residuals from the first sample also reveal obvious autocorrelation (Figure 3). Just as in simulation study 1, we can see that ACF tails off and PACF cuts off after lag 3 for GAM, suggesting AR(3) would be suitable. In contrast, the ACF and PACF of GAMAR(3) on the same data are both very close to 0. And Figure 4 indicates that the predicted spline function $\hat{ns\left(x,5\right)}$ from GAMAR(3) is much closer to the real model than that from GAM.

**Figure 5:**
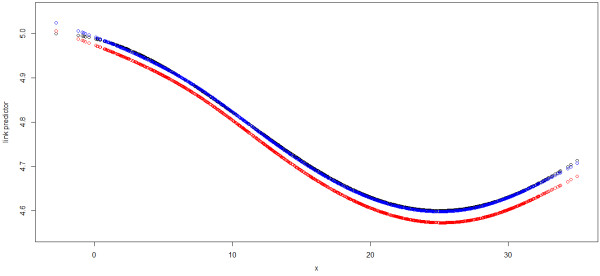
**The averaged temperature effects in link scale from Simulation study 2.** Black: the true effect, Red: $\hat{ns\left(x,6\right)}$ from GAM, Blue: $\hat{ns\left(x,6\right)}$ from GAMAR(3).

In Figure 5, we can see the mean estimated temperature effects from GAMAR(3) is closer to the true effect than that from GAM. Meanwhile, the Pearson correlation coefficients between estimated temperature effect and true effect are also different (GAM: 0.9341; GAMAR(3): 0.9980), which means GAMAR(3) provides a better fit. Also, the standard deviations of estimates from GAM are larger than those from GAMAR (Table 2).

### Application to temperature-mortality research

**Figure 6:**
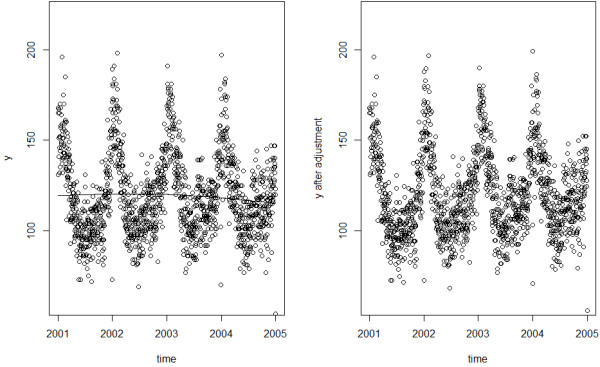
**Mortality before and after adjustment for secular trend.** Left: the original mortality, Right: the mortality adjusted for secular trend.

In the real case analysis, GAM is first used. The long time trend of original observations is unobvious as shown in the left side of Figure 6. So a rather small df: 2, is used to control this secular trend. The daily mortality after adjustment is in the right side of Figure 6.

And the complete GAM is given below:

(7) $$\begin{array}{l} E(y_{t})=\mu_{t}=\exp(\eta_{t})\\ \eta_{t}=\beta_{0}+ns(t)+ns(temp_{t-lag1})\\ \;+ns(temp_{t-lag2})+ns(pres_{t})+ns(humi_{t})\\ \;+ns(no2_{t})+ns(so2_{t})+ns(pm10_{t})\\ \;+w_{t}(week_{t}) \end{array}$$

Lagged days of two temperature terms and df of all remaining natural spline functions are first determined in a sequence to minimize AIC. Then this set of parameters is used as starting value to find the final parameters which minimize AIC locally. The final parameters are: *lag*1 = 4, *lag*2 = 10, dftemp_*t* − *lag*1_ = 5, dftemp_*t* − *lag*2_ = 4, dfpres = 2, dfhumi = 2, dfno2 = 3, dfso2 = 2, dfpm10 = 3

The two lagged temperature terms separately represent short term temperature effect and long term temperature effect. The term $w_{t}\left(week_{t}\right)=\sum_{i=1}^{6}\beta_{i}I_{i}\left(week_{t}\right)$ stands for week effect, where *week*_*t*_ represents corresponding day in a week for date t, and *I*_*i*_ (*week*_*t*_), *i* = 1,2,3,4,5,6 are indicator functions for the number of a day in a week. For each function, if *week*_*t*_ = *i, I*_*i*_ (*week*_*t*_) = 1; if *week*_t_ ≠ *i, I*_*i*_ (*week*_*t*_) = 0.

**Figure 7:**
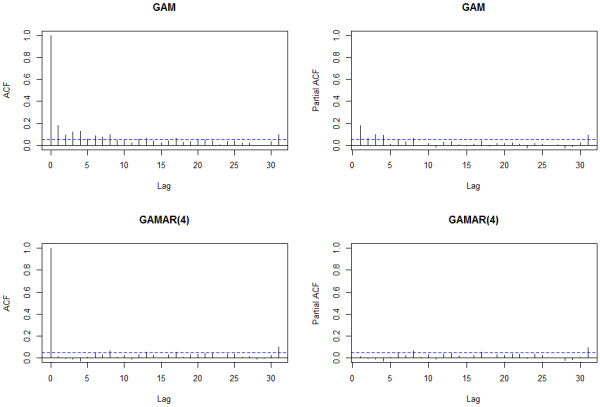
**ACF and PACF of GAM and GAMAR(4) for real case.** These statistics are calculated on Pearson residuals $z_{t}=\frac{{\hat{y}}_{t}-y_{t}}{\sqrt{{\hat{y}}_{t}}}$ from both models.

Figure 7 shows that PACF all exceed 95% CI bounds for the autocorrelations (blue dashed line) [38] lags less than 5, and are contained within the bounds for lags larger than 4. So GAMAR(4) is then chosen to fit the data as described below:

(8) $$\begin{array}{l} E(y_{t})=\mu_{t}=\exp(\eta_{t})\\ \eta_{t}=f(x_{t})+\sum_{i=1}^{4}c_{i}\left(\ln\left(y_{t-i}^{*}\right)-\eta_{t-i}\right)\\ f(x_{t})=\beta_{t}+ns(t)+ns(temp_{t-4})+ns(temp_{t-10})\\ \;+ns(pres_{t})+ns(humi_{t})+ns(no2_{t})\\ \;+ns(so2_{t})+ns(pm10_{t})+w_{t}(week_{t}) \end{array}$$

**Figure 8:**
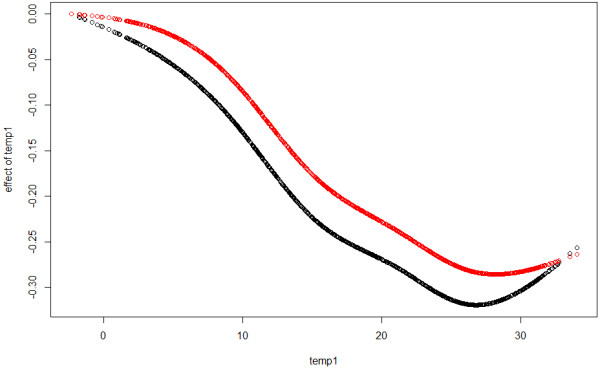
**Effects of *temp***_***t* − 4**_**.** Black: $\hat{ns\left(temp_{t-4},6\right)}$ from GAM, Red: $\hat{ns\left(temp_{t-4},6\right)}$ from GAMAR(4).

**Figure 9:**
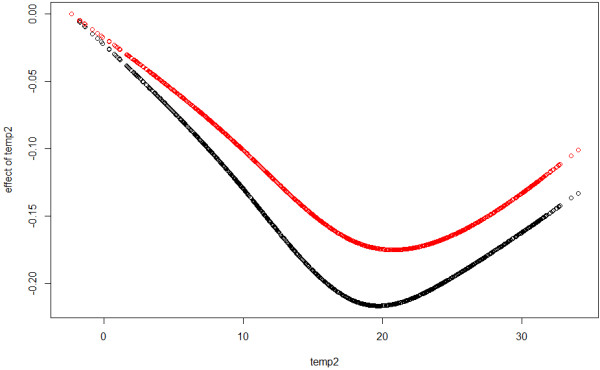
**Effects of *temp***_***t* − 10**_**.** Black: $\hat{ns\left(temp_{t-10},6\right)}$ from GAM, Red: $\hat{ns\left(temp_{t-10},6\right)}$ from GAMAR(4).

All ACF and PACF of Pearson residuals from (8) are now below 0.1, thus Pearson residuals from GAMAR(4) are close to white noise. The estimated coefficients of two lagged temperature terms and AR terms are given in Table 3. In Figure 8 and Figure 9, we can see that the estimated effects of *temp*_*t*-4_, *temp*_*t*-10_ from two models are different.

**Table 3:** temperature effects and AR estimates from GAM and GAMAR(4)

	**GAM**				**GAMAR(4)**			
	**Estimate**	**Se**	**Z**	**Pr(>|z|)**	**Estimate**	**Se**	**Z**	**Pr(>|z|)**
ns(temp1,5)1	0.2443	0.0212	−11.5349	0.0000	−0.1947	0.0254	−7.6621	0.0000
ns(temp1,5)2	0.2668	0.0281	−9.4929	0.0000	−0.2290	0.0322	−7.1108	0.0000
ns(temp1,5)3	0.3278	0.0258	−12.6878	0.0000	−0.2989	0.0290	−10.3113	0.0000
ns(temp1,5)4	0.3422	0.0495	−6.9160	0.0000	−0.2898	0.0568	−5.1052	0.0000
ns(temp1,5)5	0.2254	0.0283	−7.9578	0.0000	−0.2569	0.0320	−8.0179	0.0000
ns(temp2,4)1	0.2355	0.0197	−11.9372	0.0000	−0.1833	0.0248	−7.3986	0.0000
ns(temp2,4)2	0.1599	0.0225	−7.1233	0.0000	−0.1430	0.0263	−5.4312	0.0000
ns(temp2,4)3	0.2472	0.0443	−5.5748	0.0000	−0.1978	0.0526	−3.7603	0.0002
ns(temp2,4)4	0.0752	0.0251	−3.0007	0.0027	−0.0559	0.0297	−1.8838	0.0596
AR1					0.1426	0.0211	6.7521	0.0000
AR2					0.0773	0.0223	3.4664	0.0005
AR3					0.1179	0.0221	5.3466	0.0000
AR4					0.1259	0.0223	5.6398	0.0000

Estimate: estimate for a parameter.

Se: Standard Error for a parameter.

Z=Estimate/Se, which approximately follows N(0,1).

Pr(>|z|): the probability of obtaining Z at least as extreme as the one that was actually observed, assuming that the true value is 0. This time P value is derived from N(0,1).

## Discussion

In environmental epidemiological studies, the meteorological/environmental influences on human health indicators are often explained in a modelling framework. And the predominately used models are GLM/GAM. In our research, the drawbacks of GLM/GAM are studied, and their melioration: GAMAR, is given. Simulation studies reveal that GAMAR is more suitable when observations are autocorrelated.

While the true AR order is known in simulation studies, AR order needs to be determined in real case. Three enlightening perspectives into this issue are given below:

1. In time series analysis, ACF and PACF are good indicators of the order of process. CIs of an uncorrelated series are often treated as criterion for selecting orders [38]. For example, when PACF exceed the limit of CI at certain lag, autoregressive term at that lag needs to be modelled. This approach is used to preliminarily determine the AR order in the real case study.

2. Significance of AR terms can also be considered in modelling. If p-value of AR(n_0_+1) from GAMAR(n_0_+1) is larger than 0.05, while p-value of AR(n_0_) from GAMAR(n_0_) is less than 0.05, then GAMAR(n_0_+1) is unnecessary and GAMAR(n_0_) is a favorable model. To illustrate it, we also use GAMAR(5) to the real data, and find p-value of AR(5) to be 0.1460>0.05.

3. The goal of introducing AR terms is to control autocorrelation. So if Pearson residuals of GAMAR(n_0_) show little sign of autocorrelation, or the PACF is within the CI for all lags, then the AR terms are adequate. In real data case, this requirement is justified.

In application, the first and second advices are practical methods to determine AR order preliminarily, and the third can be used to finally justify the choice. There have been extensive discussions about choosing AR orders [39].

We also want the covariate parameter estimation to be robust with respect to different AR orders. Simulation study provides some information for this issue: while the real model is GAMAR(3) in simulation study 1. GAMAR(1), GAMAR(2), GAMAR(4), GAMAR(5) are also used for estimation. From Table 1 in Additional file we can see that GAMAR(1), GAMAR(2) fit data much better than GAM; while they differ little from GAMAR(3). Since the true AR(3) parameter is rather small (0.12), probably when the effect of neglected AR term is small, covariate parameter estimation won’t differ much. When the AR order is larger than the real, the estimation results are almost the same as GAMAR(3). In all, covariate parameter estimation is robust with respect to disturbance of AR order.

GAMM can also give good estimates as shown in Table 1. However, while the speed of GAMM in fitting short time series is acceptable, say 2.05 seconds for sample with 100 observations and 10.17 seconds for sample with 200 observations (both executed in server), the time grows nonlinearly with respect to the length of time series and thus GAMM would become computationally formidable when time series data is long. For example, one sample of 1461 observations in simulation study 1 would take about 1 hour and 7 minutes for GAMM, while only 0.02 seconds for GAMAR (both executed in server): the former is 2×10^5^ times the latter. Thus GAMM is much more computing intensive than GAMAR when sample size is large. Therefore, we only calculated 50 samples for GAMM in simulation study 1.

While we use natural cubic spline as smoother in the model, penalized spline and smoothing spline can also be implemented. With natural splines, one constructs a spline basis with knots at fixed locations throughout the range of the data. Smoothing splines and penalized splines have circumvented the problem of choosing the knot locations by constructing a very large spline basis and then penalizing the spline coefficients to reduce the effective number of df [40]. Despite the flexibility the penalized way provided, [40] identified both fully parametric and nonparametric methods can perform well in similar studies. Thus natural spline smoother can still address many practical problems. In addition, our model can be straightforwardly extended to the nonparametric way by including the penalty.

For natural spline with B-spline basis, selecting the df is of essential importance for application. A general approach is to use a data-driven method and to select the number of df which optimizes a particular criterion [40], like AIC. We used AIC to determine df of all splines except that of time in the real case. Another strategy is to use a df based on background knowledge or previous studies. For example, natural spline of time is chosen to represent the long term trend among different years, and itself shouldn’t contain any yearly fluctuation. Whether df meets the requirement is judged by comparing the trend of observations before and after adjustment, as well as the shape of the spline function visually in Figure 6. Section 2.1 of [40] gives a full treatment of this issue.

Besides including lagged temperature effects additively in the model, [41,42] have developed a family of distributed lag non-linear models (DLNM), which can simultaneously represent non-linear exposure-response dependencies and delayed effects. Still, AR terms can be aptly added to DLNM just as what we’ve done to GAM.

Finally, the model can also be applied in other areas. Researchers can use GAMAR for their specific research purposes. The most immediate extensions are other environmental epidemiological studies, specifically studies quantifying relationships between air pollution and mortality. Air pollution functions in a different way from temperature to impact human health, and there are also some differences between their modelling: 1) the air pollution-mortality relation is often simplified as linear, for such simplicity facilitates parameter estimation and interpretation; 2) distributed lag model is often used, since the effects of air pollution can last for many days; 3) cumulative effect: the total impact of pollution of a certain day over a period of following days, represented by the sum of parameters at all lags, is of great interest [9]. Such differences pose new questions for further study, like assessing the impact of AR terms on estimated cumulative effect rather than a single parameter.

## Conclusions

This article proposes GAMAR for fitting time series data with explanatory variables and autoregressive terms. Two simulation studies with functions that approximate the response from the real example showed that GAMAR performed better than GAM. In the real example, there was residual autocorrelation with GAM, but little sign of autocorrelation with GAMAR. Also, different estimates of the temperature effects were obtained with GAMAR and GAM.

## Appendix

### Modified Newton’s method

Given partial likelihood, maximum partial likelihood estimators are solved by a modified Newton’s method. For Newton’s method, the iteration goes:

(9) $$\theta_{m+1}=\theta_{m}-{\left(\frac{\partial^{2}\ln\left(PL\right)}{\partial \theta_{i}\partial \theta_{j}}\right)}^{-1}\frac{\partial \ln\left(PL\right)}{\partial \theta }|{}_{\theta =\theta_{m}}\text{,}$$

until convergence.For a modified Newton’s method, the iteration goes:

$$\theta_{m+1}=\theta_{m}+\Gamma_{*}^{-1}\left(\theta_{m}\right)\frac{\partial \ln\left(PL\right)}{\partial \theta }|{}_{\theta =\theta_{m}}\text{,}$$

until it convergence.Here $\Gamma \left(\theta \right)=-\frac{\partial^{2}\ln\left(PL\right)}{\partial \theta \partial \theta^{T}}$, which is the information matrix, and Γ_*_^− 1^(*θ*_*m*_) is a modified version of *Γ*^− 1^(*θ*_*m*_).In (9):

$$\begin{array}{l} \frac{\partial \ln\left(PL\right)}{\partial \theta_{i}}=\frac{\partial \sum_{t=1}^{n}(y_{t}\ln\left(\mu_{t}\right)-\mu_{t}-\ln\left(y_{t}!\right))}{\partial \theta_{i}}\\ \;=\sum_{t=1}^{n}\left(y_{t}-\mu_{t}\right)\frac{\partial \eta_{t}}{\partial \theta_{i}}\text{,} \end{array}$$

$$\Gamma \left(\theta \right)=-\frac{\partial \sum_{t=1}^{n}\left(y_{t}-\mu_{t}\right)\frac{\partial \eta_{t}}{\partial \theta_{i}}}{\partial \theta_{j}}=\left(\begin{array}{cc} A & B\\ B^{T} & C \end{array}\right)\text{.}$$

Since $\ln\left(\mu_{t}\right)=\eta_{t}=\sum_{i=1}^{m}\beta_{i}X_{\mathrm{ti}}+\sum_{j=1}^{p}c_{j}\left(\ln\left(y_{t-j}^{*}\right)-\sum_{i=1}^{m}\beta_{i}X_{t-j,i}\right)$, then:

$$\frac{\partial \eta_{t}}{\partial \beta_{i}}=X_{\mathrm{ti}}-\sum_{r=1}^{p}c_{r}X_{t-r,i},\frac{\partial \eta_{t}}{\partial c_{i}}=\ln\left(y_{t-i}^{*}\right)-\sum_{k=1}^{m}\beta_{k}X_{t-i,k}\text{,}$$

$$\frac{\partial^{2}\eta_{t}}{\partial \beta_{i}\partial \beta_{j}}=0,\frac{\partial^{2}\eta_{t}}{\partial c_{i}\partial c_{j}}=0,\frac{\partial^{2}\eta_{t}}{\partial \beta_{i}\partial c_{j}}=-X_{t-j,i}\text{.}$$

So

$$A={\left(\sum_{t=1}^{n}\mu_{t}(X_{\mathrm{ti}}-\sum_{r=1}^{p}c_{r}X_{t-r,i})(X_{\mathrm{tj}}-\sum_{r=1}^{p}c_{r}X_{t-r,j})\right)}_{\mathrm{mm}}\text{,}$$

$$B=\begin{array}{l} \left(\sum_{t=1}^{n},\left(\mu_{t},(,X_{\mathrm{ti}},-,\sum_{r=1}^{p}c_{r}X_{t-r,i}),\left(\ln,\left(y_{t-j}^{*}\right)\right)\right)\right)\\ \;{\left(\left(\left(-,\sum_{k=1}^{m}\beta_{k}X_{t-j,k}\right),+,\left(y_{t}-\mu_{t}\right),X_{t-j,i}\right)\right)}_{\mathrm{mp},} \end{array}$$

$$C=\begin{array}{l} \left(\sum_{t=1}^{n},\left(\ln,\left(y_{t-i}^{*}\right),-,\sum_{k=1}^{m}\beta_{k}X_{t-i,k}\right)\right)\\ {\left(\left(\ln,\left(y_{t-j}^{*}\right),-,\sum_{k=1}^{m}\beta_{k}X_{t-j,k}\right)\right)}_{\mathrm{pp}} \end{array}\text{.}$$

And the modified inverse matrix Γ_*_^− 1^(*θ*_*m*_) is defined as: if Γ(*θ*) is reversible, then Γ_*_^− 1^(*θ*) = *Γ*^− 1^(*θ*);If Γ(*θ*) is irreversible. And its eigenvalue are *λ*_1_, *λ*_2_,…,*λ*_*mp*_, then we can find orthogonal matrix *P*, which satisfies:

$$P^{T}\Gamma \left(\theta \right)P=diag\left(\lambda_{1},\ldots ,\lambda_{\mathrm{mp}}\right)\text{.}$$

Let *λ*_i_^*^ = max(*λ*_*i*_, *δ*), *δ* = 0.01, *i* = 1, 2, ⋯, *mp*, then:

$$\Gamma_{*}^{-1}\left(\theta \right)=Pdiag\left({\lambda_{1}^{*}}^{-1},\ldots ,{\lambda_{\mathrm{mp}}^{*}}^{-1}\right)P^{T}\text{.}$$

Such procedure ensures Γ_*_^− 1^(*θ*_*m*_) to be positive definite.

## Competing interests

The authors declare that they have no competing interests.

## Authors’ contributions

LY conducted the analysis and drafted the manuscript. GQ participated in discussions and provided advice. NZ inspired and tutored LY, and reviewed the paper. CW and GS collected the mortality data for real case study. All authors read and approved the final manuscript.

## Pre-publication history

The pre-publication history for this paper can be accessed here:

http://www.biomedcentral.com/1471-2288/12/165/prepub

## Supplementary Material

Additional file 1 **R code for this study.** This file contains core R codes for this study, including the function of GAMAR, data generation in simulation studies, data fitting in simulation studies, real case analysis (including the procedure to choose the parameters) and generation of tables and figures. This file also contains a brief description of every R program. Click here for file

Additional file 2 **Additional Tables.** This file contains 2 tables related to simulation study 1. Click here for file
