# Supplementary material for: Using a generalized additive model with autoregressive terms to study the effects of daily temperature on mortality
Source: BMC Med Res Methodol. 2012 Oct 30;12:165. doi: 10.1186/1471-2288-12-165 (PMC3549928; doi:10.1186/1471-2288-12-165)
Supplement: Additional file 1 — R code for this study. This file contains core R codes for this study, including the function of GAMAR, data generation in simulation studies, data fitting in simulation studies, real case analysis (including the procedure to choose the parameters) and generation of tables and figures. This file also contains a brief description of every R program. [file 1471-2288-12-165-S1.zip › R codes for this study/R codes for the study/descriptions of R files.docx]

**GAMAR.R:**

GAMAR function, fit GAMAR model and returns a list containing model coefficients.

**Choosing the degrees of freedom & real data application.R:**

Contains the procedure to choose model parameters like degrees of freedom and lags, after that, fit the model by GAM and GAMAR.

**Real data table and figures.R:**

Generate table and figures for real data analysis.

**grogam.R:**

Define function “grogam”: fit data from simulation in batch by GAM.

**grogamAR.R**

Define function “grogamAR”: fit data from simulation in batch by GAMAR.

**Simulation data analysis.R:**

Call functions “grogam” and “grogamAR” to fit data from simulations in batch, and record their estimates and CIs in batch.

**Simulation1 function.R**

Define function “simuns”: Generate data for simulation study 1 in batch.

**Simulation 1.R**

Generate data for simulation study 1 by call function “simuns”.

**Simu1Tablesfunction.R**

Define function “tablesimu”: Use the estimates and CIs in batch to generate output tables for simulation study 1.

**Simu1 tables.R**

Generate tables for simulation study 1 by “tablesimu”.

**Group analysis for gamm.R**

Fit data from simulation study 1 by GAMM, and record results one by one.

**Simu1 figures.R**

Generate figures for simulation study 1

**Simulation2 function.R**

Define function “simucos”: Generate data for simulation study 1 in batch.

**Simulation 2.R**

Generate data for simulation study 2 by call function “simucos”

**Simu2 figure.R**

Generate figures for simulation study 2

**Simu2 table.R**

Generate tables for simulation study 2
