# Supplementary material for: Using a generalized additive model with autoregressive terms to study the effects of daily temperature on mortality
Source: BMC Med Res Methodol. 2012 Oct 30;12:165. doi: 10.1186/1471-2288-12-165 (PMC3549928; doi:10.1186/1471-2288-12-165)
Supplement: Additional file 2 — Additional Tables. This file contains 2 tables related to simulation study 1. [file 1471-2288-12-165-S2.docx]

## Table 1 -Results from GAMAR(1,2,3,4,5) in simulation 1

TruPar=True Parameters=$\beta_{i}$ or $c_{i}$

MeaEst=Mean estimates=$\bar{\hat{\beta}}_{i}$ or $\bar{\hat{c}}_{i}$

Bias=$\bar{\hat{\beta}}_{i}-\beta_{i}$or $\bar{\hat{c}}_{i}-c_{i}$

RelErr=Relative Error=$\overline{\left|(\hat{\beta}_{i}-\beta_{i})/\beta_{i}\right|}$or $\overline{\left|(\hat{c}_{i}-c_{i})/c_{i}\right|}$

Coverage:the percentage ofestimated 95%CI which covers true coefficient in all estimated 95%CI.

Mea_co: Mean of absolute Bias, RelErr, Coverage for parameters of covariates.

Mea_ar: Mean of absolute Bias, RelErr, Coverage for parameters of AR terms.

|  | |  | GAMAR(1) | | | | GAMAR(2) | | | | GAMAR(3) | | | |
| --- | --- | --- | --- | --- | --- | --- | --- | --- | --- | --- | --- | --- | --- | --- |
|  | TruPar | | MeaEst | Bias | RelErr | Coverage | MeaEst | Bias | RelErr | Coverage | MeaEst | Bias | RelErr | Coverage |
| $\beta_{0}$ | 5.02 | | 5.0085 | -0.0115 | 0.0063 | 89.8 | 5.0149 | -0.0051 | 0.0055 | 94.3 | 5.0182 | -0.0018 | 0.0055 | 94.8 |
| $\beta_{1}$ | -0.35 | | -0.3563 | -0.0063 | 0.0802 | 93.4 | -0.3562 | -0.0062 | 0.0696 | 94.8 | -0.3561 | -0.0061 | 0.0686 | 93.9 |
| $\beta_{2}$ | -0.36 | | -0.3641 | -0.0041 | 0.1072 | 90.2 | -0.3626 | -0.0026 | 0.0904 | 94 | -0.362 | -0.002 | 0.0885 | 94.6 |
| $\beta_{3}$ | -0.38 | | -0.3782 | 0.0018 | 0.0867 | 88 | -0.3765 | 0.0035 | 0.0739 | 93.3 | -0.3757 | 0.0043 | 0.0733 | 93.8 |
| $\beta_{4}$ | -0.33 | | -0.3286 | 0.0014 | 0.1865 | 94.6 | -0.3267 | 0.0033 | 0.1606 | 95.5 | -0.3262 | 0.0038 | 0.1604 | 95.5 |
| $\beta_{5}$ | -0.15 | | -0.1489 | 0.0011 | 0.2564 | 87.5 | -0.1474 | 0.0026 | 0.2234 | 93.3 | -0.1472 | 0.0028 | 0.2201 | 93.8 |
|  | Mea_co | |  | 0.0044 | 0.1206 | 90.6 |  | 0.0039 | 0.1039 | 94.2 |  | 0.0035 | 0.1027 | 94.4 |
| $c_{1}$ | 0.5 | | 0.7786 | 0.2786 | 0.5572 | 0 | 0.5358 | 0.0358 | 0.074 | 70.5 | 0.4982 | -0.0018 | 0.0422 | 95.3 |
| $c_{1}$ | 0.25 | |  |  |  |  | 0.3118 | 0.0618 | 0.2475 | 29.5 | 0.2477 | -0.0023 | 0.0947 | 93.4 |
| $c_{1}$ | 0.12 | |  |  |  |  |  |  |  |  | 0.1197 | -0.0003 | 0.1737 | 94.7 |
|  | Mea_ar | |  | 0.2786 | 0.5572 | 0 | 0.4238 | 0.0488 | 0.1608 | 50 |  | 0.0015 | 0.1035 | 94.5 |
|  | |  |  |  |  |  |  |  |  |  |  |  |  |  |

|  |  | GAMAR(4) | | | | GAMAR(5) | | | |
| --- | --- | --- | --- | --- | --- | --- | --- | --- | --- |
|  | TruPar | estimators | Bias | R err | coverage | estimators | Bias | R err | coverage |
| $\beta_{0}$ | 5.02 | 5.0181 | -0.0019 | 0.0055 | 94.9 | 5.018 | -0.002 | 0.0055 | 95.2 |
| $\beta_{1}$ | -0.35 | -0.356 | -0.006 | 0.0688 | 93.8 | -0.356 | -0.006 | 0.069 | 94 |
| $\beta_{2}$ | -0.36 | -0.362 | -0.002 | 0.0887 | 94.7 | -0.3619 | -0.0019 | 0.0888 | 94.7 |
| $\beta_{3}$ | -0.38 | -0.3757 | 0.0043 | 0.0735 | 94 | -0.3757 | 0.0043 | 0.0735 | 93.9 |
| $\beta_{4}$ | -0.33 | -0.326 | 0.004 | 0.1609 | 95.4 | -0.3259 | 0.0041 | 0.1611 | 95.8 |
| $\beta_{5}$ | -0.15 | -0.1472 | 0.0028 | 0.2205 | 93.7 | -0.1472 | 0.0028 | 0.2206 | 93.8 |
|  | Mea_co |  | 0.0035 | 0.1030 | 94.4 | 0.5752 | 0.0035 | 0.1030 | 94.4 |
| $c_{1}$ | 0.5 | 0.4983 | -0.0017 | 0.0427 | 95 | 0.4983 | -0.0017 | 0.0428 | 95.1 |
| $c_{1}$ | 0.25 | 0.2482 | -0.0018 | 0.0974 | 94.2 | 0.2485 | -0.0015 | 0.0978 | 94.1 |
| $c_{1}$ | 0.12 | 0.1205 | 0.0005 | 0.1923 | 95.6 | 0.1211 | 0.0011 | 0.1992 | 95.8 |
|  | Mea_ar |  | 0.0013 | 0.1108 | 94.9 |  | 0.0013 | 0.1108 | 94.9 |
|  | 0 | -0.0016 | -0.0016 |  | 95.4 | -0.0005 | -0.0005 |  | 95.7 |
|  | 0 |  |  |  |  | -0.0023 | -0.0023 |  | 94.9 |
|  |  |  |  |  |  |  |  |  |  |

## Table 2 -Results from GAMAR(3) for different time range in simulation 1

TruPar=True Parameters=$\beta_{i}$ or $c_{i}$

MeaEst=Mean estimates=$\bar{\hat{\beta}}_{i}$ or $\bar{\hat{c}}_{i}$

Bias=$\bar{\hat{\beta}}_{i}-\beta_{i}$or $\bar{\hat{c}}_{i}-c_{i}$

RelErr=Relative Error=$\overline{\left|(\hat{\beta}_{i}-\beta_{i})/\beta_{i}\right|}$or $\overline{\left|(\hat{c}_{i}-c_{i})/c_{i}\right|}$

Coverage:the percentage ofestimated 95%CI which covers true coefficient in all estimated 95%CI.

Mea_co: Mean of absolute Bias, RelErr, Coverage for parameters of covariates.

Mea_ar: Mean of absolute Bias, RelErr, Coverage for parameters of AR terms.

|  |  | 2y | | | | 4y | | | | 6y | | | |
| --- | --- | --- | --- | --- | --- | --- | --- | --- | --- | --- | --- | --- | --- |
|  | TruPar | MeaEst | Bias | RelErr | Coverage | MeaEst | Bias | RelErr | Coverage | MeaEst | Bias | RelErr | Coverage |
| $\beta_{0}$ | 5.02 | 5.0101 | -0.0099 | 0.0077 | 92 | 5.0182 | -0.0018 | 0.0055 | 94.8 | 5.0193 | -0.0007 | 0.0046 | 94 |
| $\beta_{1}$ | -0.35 | -0.3521 | -0.0021 | 0.092 | 94.1 | -0.3561 | -0.0061 | 0.0686 | 93.9 | -0.3529 | -0.0029 | 0.0572 | 93.7 |
| $\beta_{2}$ | -0.36 | -0.3537 | 0.0063 | 0.1248 | 93.6 | -0.362 | -0.002 | 0.0885 | 94.6 | -0.3625 | -0.0025 | 0.0702 | 94.6 |
| $\beta_{3}$ | -0.38 | -0.364 | 0.016 | 0.1107 | 92.1 | -0.3757 | 0.0043 | 0.0733 | 93.8 | -0.3777 | 0.0023 | 0.0602 | 94.8 |
| $\beta_{4}$ | -0.33 | -0.3579 | -0.0279 | 0.2205 | 92.4 | -0.3262 | 0.0038 | 0.1604 | 95.5 | -0.327 | 0.003 | 0.1317 | 95.4 |
| $\beta_{5}$ | -0.15 | -0.1699 | -0.0199 | 0.3004 | 92.2 | -0.1472 | 0.0028 | 0.2201 | 93.8 | -0.148 | 0.002 | 0.1883 | 94.5 |
|  | Mea_co |  | 0.0137 | 0.1427 | 92.7 |  | 0.0035 | 0.1027 | 94.4 |  | 0.0022 | 0.0854 | 94.5 |
| $c_{1}$ | 0.5 | 0.4952 | -0.0048 | 0.0597 | 95.4 | 0.4982 | -0.0018 | 0.0422 | 95.3 | 0.4989 | -0.0011 | 0.034 | 94.8 |
| $c_{1}$ | 0.25 | 0.2458 | -0.0042 | 0.1337 | 94.6 | 0.2477 | -0.0023 | 0.0947 | 93.4 | 0.2486 | -0.0014 | 0.0763 | 94.6 |
| $c_{1}$ | 0.12 | 0.1185 | -0.0015 | 0.248 | 95.2 | 0.1197 | -0.0003 | 0.1737 | 94.7 | 0.1198 | -0.0002 | 0.1364 | 94.9 |
|  | Mea_ar |  | 0.0035 | 0.1471 | 95.1 |  | 0.0015 | 0.1035 | 94.5 |  | 0.0009 | 0.0822 | 94.8 |
|  |  |  |  |  |  |  |  |  |  |  |  |  |  |
